# Impact of COVID-19 pandemic on chronic pain and opioid use in marginalized populations: A scoping review

**DOI:** 10.3389/fpubh.2023.1046683

**Published:** 2023-04-17

**Authors:** Karen Choe, Eleanor Zinn, Kevin Lu, Dung Hoang, Lawrence H. Yang

**Affiliations:** ^1^School of Global Public Health, New York University, New York, NY, United States; ^2^Teachers College Columbia University, New York, NY, United States; ^3^Mailman School of Public Health, Columbia University, New York, NY, United States

**Keywords:** chronic nonmalignant pain, COVID-19, opioid use, addiction, pain management

## Abstract

**Introduction:**

The COVID-19 pandemic has had a variable effect on vulnerable populations, including patients with chronic pain who rely on opioid treatment or have comorbid opioid use disorder. Limited access to care due to isolation measures may lead to increased pain severity, worse mental health symptoms, and adverse opioid-related outcomes. This scoping review aimed to understand the impact of the COVID-19 pandemic on the dual epidemics of chronic pain and opioids in marginalized communities worldwide.

**Methods:**

Searches of primary databases including PubMed, Web of Science, Scopus, and PsycINFO were performed in March 2022, restricting the publication date to December 1, 2019. The search yielded 685 articles. After title and abstract screening, 526 records were screened by title and abstract, 87 through full-text review, of which 25 articles were included in the final analysis.

**Results:**

Our findings illuminate the differential distribution of pain burden across marginalized groups and how it serves to heighten existing disparities. Service disruptions due to social distancing orders and infrastructural limitations prevented patients from receiving the care they needed, resulting in adverse psychological and physical health outcomes. Efforts to adapt to COVID-19 circumstances included modifications to opioid prescribing regulations and workflows and expanded telemedicine services.

**Conclusion:**

Results have implications for the prevention and management of chronic pain and opioid use disorder, such as challenges in adopting telemedicine in low-resource settings and opportunities to strengthen public health and social care systems with a multidisciplinary and multidimensional approach.

## 1. Introduction

### 1.1. COVID-19, chronic pain, and opioids

Since the end of 2019, Coronavirus (COVID-19), an infectious disease caused by severe acute respiratory syndrome coronavirus 2 (SARS-CoV-2), has placed the world under unprecedented circumstances. Rapidly spreading amongst the human population, the pandemic has severely impacted major economies, job markets, and societies (1). With the implementation of shelter-in-place quarantine measures, individuals infected with and those vulnerable to COVID-19 have experienced isolation and worsened mental health symptoms (e.g., anxiety and depressive disorders, substance use disorders). The lockdown has limited access to life opportunities, disproportionately affecting marginalized communities, including low-income and minority groups who have experienced higher COVID-related mortality rates and hospitalization (2). In particular, patients with chronic pain who rely on opioid treatment are faced with limited access to assessment and intervention due to the pandemic, potentially leading to a substantial increase in pain severity and opioid-related deaths, compounding the already trenchant public health issue of opioid use and chronic pain (3).

### 1.2. Opioid epidemic

Substance use is a leading contributor to premature deaths worldwide, with drug overdoses ranking high among the leading causes of death in many countries (4, 5). Over 70% of the 5.5% of global deaths attributed to substance use disorders are due to opioids, with over 30% resulting from overdose – most of which could have been prevented (6, 7).

**Figure 1:**
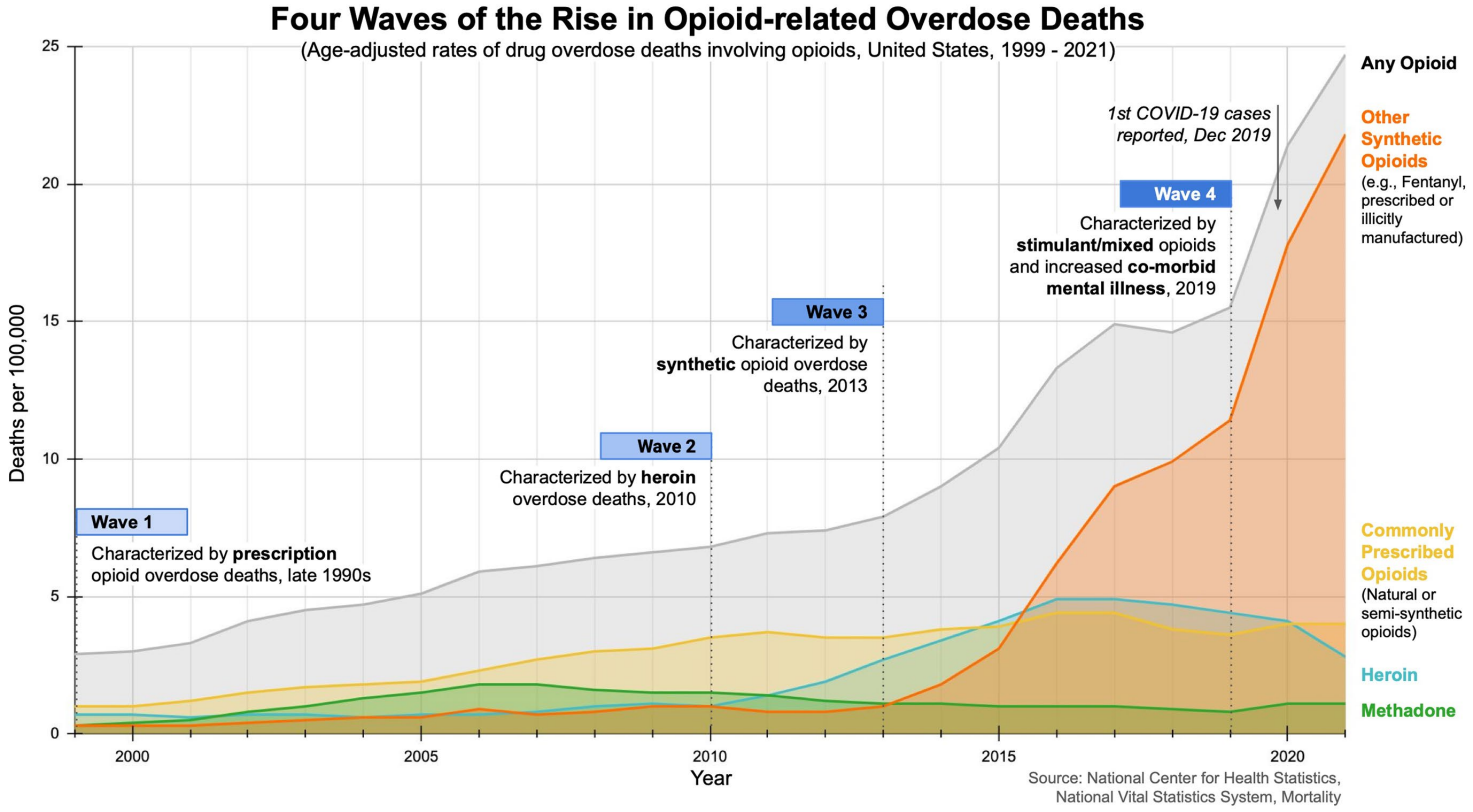
Four waves of the rise in opioid-related overdose deaths.

The opioid epidemic is a pressing global public health crisis, driven by the rise of improper prescription practices, opioid misuse, and undertreatment of pain (8, 9). The crisis, marked by high disability and mortality rates, has had a profound impact on communities worldwide (10). The opioid crisis has unfolded in three distinct waves, with recent data suggesting a fourth wave (see Figure 1). The first wave of opioid overdose deaths can be traced back to the 1990s, when the usage and availability of pharmaceutical opioids began to increase (11, 12). Opioids were initially prescribed for short-term use (e.g., pain relief after surgery). In 1995, Purdue Pharma introduced and heavily promoted OxyContin as a safe, effective treatment for chronic pain (11, 12). At the time, pharmaceutical companies falsely assured medical communities that opioid pain relievers were not addictive, leading to higher prescription rates and increased risk for illicit drug use. The second wave, marked by a rise in heroin-related overdose deaths, began in 2010. By 2012, the total number of prescriptions dispensed peaked at 255 million, with 81.3 prescriptions per 100 individuals in the US. The third wave, characterized by overdoses attributed to synthetic opioids, started in 2013 (11, 12). In 2017, an estimated 40.5 million people were dependent on opioids, with 80% of all opioid prescriptions written globally in the US, prompting the US Department of Health & Human Services to declare a public health emergency and develop a five-point strategy (4, 13, 14). Emerging data suggest a fourth wave characterized by overdose deaths involving illicit fentanyl, combined use of opioids and stimulants (cocaine, methamphetamine), exacerbated by co-morbid mental illness and the COVID-19 pandemic (15–18).

### 1.3. Chronic nonmalignant pain

Chronic nonmalignant pain is defined as pain that persists longer than three to six months beyond the usual course of disease or injury (19). It is a multidimensional phenomenon with physical, psychological, and sociocultural impacts on health (20). In the US, about one in five, or 50 million people, experience chronic pain. Among those, 36.4% live with high-impact chronic pain that significantly limits life or work activities, with women (8.5%) and adults aged 65 and over (11.8%) being the most affected. The prevalence of chronic pain has increased for all adult demographics but has seen the most significant increases in older adults and those with low socioeconomic status (SES) (21). Globally, the prevalence of chronic pain ranged from 10.1 to 40.0%, with a weighted mean prevalence of 30.3% in 2021 (22).

Chronic pain is a primary reason adults seek medical care, and is associated with poor mental health, opioid dependence, and decreased quality of life (21). Chronic pain can arise from various conditions, some with unknown causes, and manifest differently across individuals. Managing chronic pain can be challenging due to its complexity, but prescription medication, physical therapy, and surgical implants are common interventions (8). For those who are unresponsive to these approaches or suffer from moderate to severe chronic noncancer pain, a combination of long-term opioid therapy (LTOT) and self-management treatment modalities may be recommended (23, 24).

### 1.4. Opioids in the context of chronic pain

Opioids and chronic pain are intimately linked, as opioid medications are among the most common pain treatments (25). Despite benefits in alleviating pain, opioids have highly addictive properties, with the risk for overdose increasing with dosage (such as to counter the effects of opioid-induced hyperalgesia) and period of use (26, 27). Although prescription rates vary, an estimated 20–22% of US adults with chronic pain used prescription opioids for their chronic pain in 2019 (28). Pain is commonly cited as a potential precursor to opioid use disorder (OUD), but the prevalence of prescription opioid abuse among patients with chronic pain is unclear (24, 29, 30). Studies suggest that 14–19% of patients who receive prescription opioids struggle with opioid dependence (14), and less than 3% of these patients develop OUD (31). Treatment for OUD consists of medications (e.g., methadone, buprenorphine, and naltrexone) to reduce withdrawal symptoms and eliminate cravings, as well as psychosocial support, counseling, and social services targeted toward community reintegration (32, 33).

### 1.5. Biopsychosocial barriers to care and marginalized groups

The COVID-19 pandemic highlights the importance of the biopsychosocial model, which recognizes the unique interaction of biological, psychological, and social factors impacting each individual’s experience of pain and disability (34). Health inequities experienced by marginalized groups are rooted in socioeconomic determinants, are socially produced (and therefore, modifiable), and can vary within populations (35). In the absence of a universal definition in the literature, we used the following definition of marginalized populations for the current review: “groups and communities that experience discrimination and exclusion (social, political and economic) because of unequal power relationships across economic, political, social and cultural dimensions.” These include, but are not limited to, racial and ethnic minority populations (including Blacks/African-Americans, Hispanics/Latinos, Asians, Pacific Islanders, American Indians, and Alaska Natives), low-to lower middle-income populations, underserved and/or rural populations, sexual and gender minorities, and people with disabilities (36–40).

Chronic pain and OUD are linked to biopsychosocial barriers to care that include limited access to medications, chronic unemployment, poverty, mental illness, stigma, social isolation, and poor social support (41). Socioeconomic marginalization is positively associated with overdose and prevalence rates (42). People of lower SES often hold more physically demanding jobs that exacerbate chronic pain (43) and bear a disproportionate economic burden of chronic pain (44–46). Financial strains amplify the risk of higher pain severity, pain-related disability (47–49), limited access to opioid-related care, misuse, diversion, and fatal overdose (50–52).

The experience and communication of pain can vary among marginalized groups. For example, Black and Hispanic Americans experience greater pain severity and related physical and psychosocial disability than non-Hispanic whites but are more likely to underreport their pain to primary care providers (PCPs) (53–57). Concerns around seeking and receiving appropriate pain treatment often point to the stigmatization of opioid use in marginalized populations (58). Researchers have also examined disparate treatment practices and outcomes, such as ethnic/racial and socioeconomic disparities in opioid prescriptions (56, 59). Clinicians may underestimate pain intensity and carry varying expectations about the risk of opioid misuse among different groups, leading to inadequate pain management, reduced analgesic prescriptions, and differential access to evidence-based treatment options (60–63). Investigating potential disparities in access to and experiences of care, particularly with the advent of telehealth during the pandemic, may provide vital information for future efforts to improve health equity.

Social and economic inequities not only pose barriers to accessing healthcare but induce and exacerbate chronic pain and opioid use (64–67). Emerging data has shown that the pandemic has coincided with a spike in opioid overdose deaths and worsened chronic pain outcomes, largely due to limited access to treatment (68, 69). Even prior to the pandemic, a significant proportion of people with chronic pain (estimated 40 to 70%) did not receive adequate medical care (14). Undiagnosed or inadequately treated pain can result in mental health consequences, with patients experiencing three times the risk of depression and anxiety and at least twice the risk of suicide than those without chronic pain (70, 71). The high prevalence of chronic pain and increasing use of opioids, resulting in a ‘silent epidemic,’ highlight the need for effective and harm-minimizing approaches to address pain.

During the pandemic, individuals have faced compounding issues from reduced access to crucial avenues of assessment and treatment, in addition to disruptions in social service programs, job losses, housing instabilities, increases in family responsibilities, fewer caretakers due to infection risks, and food insecurities (34, 72). These disruptions likely lead to downstream effects that increase dependence and relapse risk and undermine long-term recovery (73). However, it remains unclear as to what extent the pandemic has affected patients with chronic pain who use opioids from marginalized populations. Preliminary literature searches have found a limited number of reviews on the intersection of chronic pain and opioid use. Few studies have assessed risk for developing opioid-related adverse outcomes (e.g., overdose) or marginalization (e.g., managing low or lost income, mental illness, housing instability, social isolation) during the pandemic. Therefore, this scoping review aims to investigate the intersection of opioid use and opioid-related services, chronic pain, and COVID-19 globally, focusing on marginalization from endemic structural disparities in healthcare. By reviewing the consequences of concurrent epidemics on chronic pain, opioid use, and COVID-19, we aim to enhance our understanding of social marginalization and health inequalities between and within populations and identify ways to strengthen public health and social care systems.

## 2. Methods

A scoping review was conducted through the Joanna Briggs Institute Reviewers’ Manual 2015 Methodology for JBI Scoping Reviews (74). A scoping review was most appropriate for meeting our current study objectives, addressing broad research questions, and summarizing the available literature. We examined the published literature relevant to our research questions, identified gaps in knowledge, and reported per the guidance provided in the Preferred Reporting Items for Systematic Reviews and Meta-Analyses Protocols (PRISMA-P) (75).

### 2.1. Inclusion and exclusion criteria

Publications that met the inclusion criteria focused on patients with chronic pain who use opioids, included marginalized group(s), included the context of the COVID-19 pandemic, and were published from December 1, 2019 to March 1, 2022. All primary research studies, reviews, meta-analyses, guidelines, texts published on society websites, editorials, and comments/commentaries were considered. We considered articles on various intersecting groups, including but not limited to patients with chronic pain who use opioid analgesics on a long-term basis, people with pain who are receiving medications for their OUD, and those who experience both OUD and chronic pain untreated. In addition to the definition of ‘marginalization’ previously mentioned, we recognize ‘marginalization’ is an umbrella term and thus considered articles using the terms ‘seldom heard,’ ‘hard to reach,’ and ‘vulnerable.’ These terms have been used in literature to describe groups who experience barriers to accessing services, have little to no voice in healthcare policy planning and/or resource allocation, and/or may experience barriers to communicating health needs (due to impairment, personal context, or consequence of stigma) (76).

Publications that addressed acute pain, cancer pain, or chronic pain outside the context of the COVID-19 pandemic were excluded. Articles in languages other than English, articles that did not have full text available, protocol papers, case studies, non-peer-reviewed articles, and dissertations were also excluded.

### 2.2. Search strategy

PubMed, Web of Science, Scopus, and PsycINFO databases were searched using a comprehensive search strategy. An initial search was carried out in 2020, followed by an analysis of the words contained in the title, abstract, and index to describe the article. A complete secondary search was performed in March 2022 on all databases, using the terms identified in the initial limited search (see Table 1 for the scientific literature search strategy). The search yielded 685 articles. After duplicates were removed, 526 remaining abstracts were screened against the inclusion criteria.

**Table 1:** Scientific literature search strategy.

Databases Searched	Search terms
PubMed, Web of Science, Scopus, and PsycINFO	Coronavirus Infections; COVID-19; 2019 novel coronavirus disease; COVID19; COVID-19 pandemic; SARS-CoV-2 infection; COVID-19 virus disease; 2019 novel coronavirus; 2019-nCoV infection; coronavirus disease 2019; coronavirus disease-19; 2019-nCoV disease; COVID-19 virus infection; Coronavirus Infection; Infection, Coronavirus; Infections, Coronavirus; Middle East Respiratory Syndrome; MERS; opioid use disorder; Opioids; Opioid; opioid treatment; addiction; substance use disorder; substance use; buprenorphine; heroin; opioid therapy; morphine; fentanyl; addict; opiate addiction; people who use drugs; narcotic; prescription abuse; prescription opioid abuse; naloxone; prescribing opioids; opioid dependence; chronic pain; Pains, Chronic; Pain, Chronic; Widespread Chronic Pain; Chronic Pain, Widespread; Chronic Pains, Widespread; Pain, Widespread Chronic; Pains, Widespread Chronic; Widespread Chronic Pains; pain management

**Figure 2:**
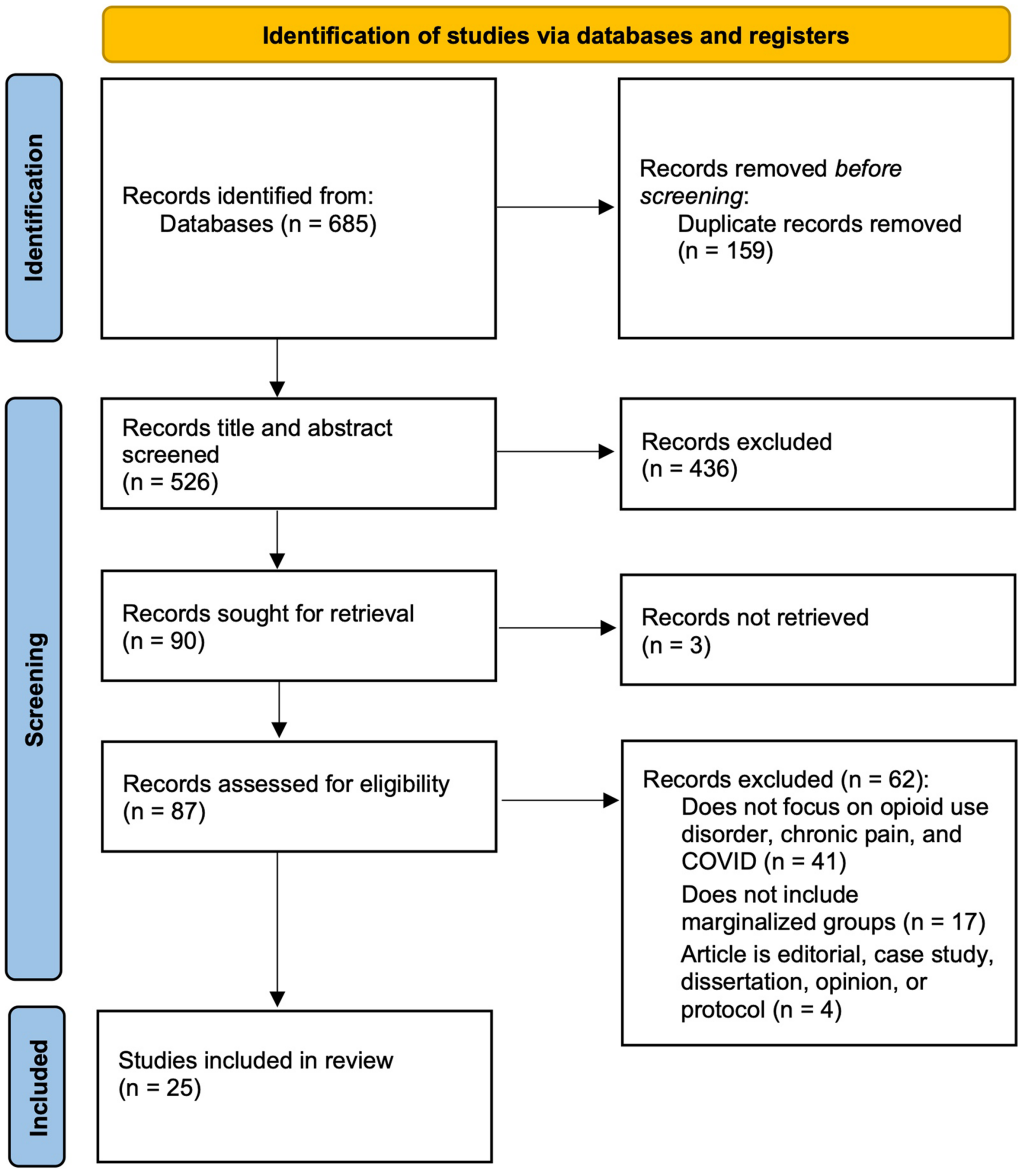
PRISMA flow diagram.

The reviewers developed a database using excel to confirm relevance and extract key study characteristics, including (1) publication year, (2) publication type, (3) methodology, (4) country, (5) economic level [as classified by World Bank (77)], (6) population (e.g., specific marginalized group[s]). Each abstract was screened and examined by two independent reviewers based on the inclusion criteria. 87 out of 90 articles were retrieved for full-text review. Disagreements between reviewers at each stage of the study selection process were resolved through discussion or with a third reviewer. Using Cohen’s Kappa, inter-rater reliability was calculated to be 0.777, indicating substantial agreement. 25 full-text articles met inclusion criteria and were included for final synthesis. After the searches, all citations were organized in a reference manager. The results are presented in a PRISMA-ScR flow diagram (see Figure 2). The extracted data (*N* = 25) includes the distribution of studies by year, author, title, type of publication, and content and are presented in tables (see Tables 2, 3) and narrative summary.

**Table 2:** Characteristics of included publications.

Article	Publication type	Country of focus; World bank income level	Content	Population (Marginalized groups of focus)
Akhtar, 2020 (78)	Editorial view	Pakistan; Lower middle	Discusses interventional pain services and shift to telemedicine	Patients with CP who use opioids (Multiple; SES, geographic location, race/ethnicity)
Ayad, 2021 (79)	Brief communication	Global (United States, Armenia, Middle Eastern countries, Vietnam, Mexico, India, etc.) with focus on Egypt; Lower middle	Reflections on the burden of CP and the opioid epidemic in developing countries	Patients with CP who use opioids (Multiple; SES, geographic location)
Chan, 2020 (80)	Commentary	Singapore; High	Discusses decisions and safeguards made at a tertiary pain center; recommendations to guide decision-making in pain management	Patients with CP who use opioids (Elderly)
Cohen, 2020 (9)	Expert opinion	United States; High	Framework for pain practitioners/institutions to balance the often conflicting goals of risk mitigation for healthcare providers, risk mitigation for patients, conservation of resources, and access to pain management services	Patients with CP who use opioids (Multiple; Elderly, Disability)
Compton, 2022 (81)	Review	United States; High	Review of pandemic-related stressors on problematic substance and opioid use and how COVID-19 infection and debilitation may worsen experiences of CP	Patients with CP and OUD (SES, geographic location, race/ethnicity)
de Moraes, 2021 (82)	Scoping Review	Global (English-speaking countries, Latin countries); Mixed	Recommendations on the workflow and management of patients with CP during the COVID-19 pandemic	Patients with CP who use opioids (*N* = 13 studies) (Multiple; Race/ethnicity)
Dunn, 2021 (83)	Original research	United States; High	RCT; evaluation of feasibility and acceptability of electronic and cellular-enabled pillbox to deliver split-doses of methadone for treatment of OUD among patients with persistent pain	Patients with OUD and persistent pain who receive methadone (*N* = 25) (Multiple; Race/ethnicity, disability)
Edmond, 2021 (84)	Original research	United States; High	Evaluation of adaptations made by interdisciplinary pain team to deliver services virtually, which include (1) individualized assessment of LTOT; (2) reduction or discontinuation of LTOT when benefits no longer outweigh the harms; (3) switch to buprenorphine if difficulty tapering or OUD emerges; (4) optimization of virtual treatment	Patients with CP on LTOT (*N* = 29) (Race/ethnicity)
El-Tallawy, 2020 (85)	Review	United States; High	Review of changes in healthcare delivery during the COVID-19 pandemic; provides guidance on pain management and concentrating available resources to help patients with most severe conditions and for most vulnerable groups	Patients with CP who use opioids (Multiple; Elderly)
George, 2020 (86)	Original research	Singapore; High	Discusses implementation of integrating a pain center with community healthcare teams to mitigate harmful effects of treatment disruption during the pandemic for vulnerable individuals with CP and comorbid conditions	Vulnerable older patients with CP and multiple comorbidities who use opioids (Multiple; Elderly, disability)
Humphreys, 2022 (87)	Review	United States, Canada; High	Empirically grounded analysis of the causes of, and solutions, to the opioid crisis, including proposed domestic solutions and attempts to stop international spread	Patients with CP who use opioids (Multiple; SES, race/ethnicity)
Joyce, 2020 (88)	Original research	United States; High	Details the effects of the early-stage COVID-19 pandemic on interventional pain physicians’ decision-making, practice patterns, and mental health	Physicians who work with patients with CP and prescribe opioids (*N* = 260) (Geographic location, disability [High risk])
Katzman, 2021 (89)	Commentary	United States; High	Reflections on how COVID-19 has highlighted multiple epidemics, including CP, substance use disorder, gun violence, suicide, and loneliness, disproportionately impacting marginalized communities	Patients with CP who use opioids (Multiple)
Lee, 2021 (90)	Original research	United States; High	Retrospective, cross-sectional study to assess trends in opioid prescription and nonpharmacologic therapy (e.g., physical therapy, complementary medicine) for pain management during early months of the COVID-19 pandemic compared with patterns in 2019	Claims data from patients with varying CP diagnoses (*N* = 21,430,339) (Multiple; SES, race/ethnicity, elderly, disability)
Licciardone, 2021a (91)	Original research	United States; High	Pre-post study; Assesses changes in patients’ use of nonpharmacological and pharmacological treatments for chronic low back pain and related outcomes during the COVID-19 pandemic	Patients with chronic low back pain (*N* = 476)(Multiple; Race/ethnicity, elderly)
Licciardone, 2021b (92)	Original research	United States; High	Pre-post study; Examines how reduced access to care impacted use of recommended nonpharmacological treatments, nonsteroidal anti-inflammatory drugs, and opioids and clinical outcomes among patients with chronic low back pain	Patients with chronic low back pain (*N* = 528)(Multiple; Race/ethnicity, elderly, disability)
Manchikanti, 2021 (3)	Review	Global (US, Australia, El Salvador, Canada, Uruguay, Iceland, Sweden, New Zealand, Finland, UK) with focus on US; Mostly high	Exploration of opioid and COVID-19 epidemics and improvements needed in access to the management of CP with physical therapy, interventional techniques, appropriate diagnostic modalities, and opioid therapy	Patients with CP who use opioids(Multiple)
Morgan, 2021 (93)	Review	United States; High	Multidisciplinary project involving 49 clinic sites designed to implement and evaluate clinic-based interventions to improve pain management, reduce harms from opioid overprescribing, and treatment of OUD during COVID in a predominantly Medicaid population	Patients with CP and OUD (SES; Medicaid, Medicare)
Mun, 2021 (94)	Original research	United States; High	Cross-sectional survey assessing impact of COVID-19 on pain severity and management, factors associated with perceived changes in pain severity	Patients with CP (*N* = 1,453)(Race/ethnicity)
Mun, 2022 (95)	Original research	United States; High	Longitudinal 1-year study that examines impact of COVID-19 pandemic on trajectories of pain severity, interference, emotional distress (i.e., anxiety and depressive symptoms), and opioid misuse behaviors	Patients with CP (*N* = 1,453; 878; 813 over 3 surveyed periods)(SES, race/ethnicity)
Oh et al., 2021 (96)	Original research	South Korea; High	Population-based retrospective cohort study; examined in-hospital mortality rates among patients with musculoskeletal disorders who take pain medications (NSAIDs, strong opioids, weak opioids) and who tested positive for COVID-19	Patients with musculoskeletal disorders, COVID-19, and identified as long-term opioid users (*N* = 7,713) (Multiple: SES, geographic location, elderly, disability)
Prater, 2020 (97)	Original research	United States; High	Pilot prospective observational study that aims to improve systems on spinal pain treatment within primary medical homes	Underserved patients with chronic spinal pain (*N* = 35)(SES: Low income and uninsured, race/ethnicity)
Rao, 2020 (98)	Commentary	United States; High	Highlights evidence and best practices in telehealth in pain management, including comprehensive and effective opioid management	Patients with CP with opioid therapy(Multiple; Elderly)
Shanthanna, 2020 (99)	Expert opinion	Global (United States, Europe); Mixed	Recommendations by expert model for doctors and healthcare professionals to guide practice of CP management during period of crisis	Patients with CP with opioid therapy(Multiple; Elderly)
Tuan, 2021 (100)	Original research	United States; High	Retrospective cohort study to assess risk of developing severe clinical outcomes among COVID-19 noncancer patients on LTOT, compared with those without LTOT	Adult patients (*N* = 418,216) with COVID-19; treated with LTOT for CP (*N* = 9,558)(Multiple; SES, race/ethnicity, elderly)

NR, not reported; OUD, opioid use disorder; LTOT, long-term opioid therapy; CP, chronic pain; SES, socioeconomic status.

**Table 3:** Participant characteristics of included original research articles.

Article	Participants	Mean age (SD)	Sex
Dunn, 2021 (83)	Patients with OUD and persistent pain who receive methadone (*N* = 25)	33 (10.7)	42.3% M, 57.7% F
Edmond, 2021 (84)	Patients with CP on LTOT (*N* = 29)	62.9 (11.2)	86.2% M, 13.8% F
George, 2020 (86)	Vulnerable older patients with CP and multiple comorbidities who use opioids	58	NR
Joyce, 2020 (88)	Physicians who work with patients with CP and prescribe opioids (*N* = 260)	48.6 (10.0)	86% M, 14% F
Lee, 2021 (90)	Claims data from patients with varying CP diagnoses (*N* = 21,430,339)	48.6 (24.0)	48.9% M, 51.1% F
Licciardone, 2021a (91)	Patients with chronic low back pain (*N* = 476)	54 (13.2)	26.7% M, 73.3% F
Licciardone, 2021b (92)	Patients with chronic low back pain (*N* = 528)	53.9 (13.0)	25.9% M, 74.1% F
Mun, 2021 (94)	Patients with CP (*N* = 1,453)	41.7 (13.1)	34.8% M, 65.2% F
Mun, 2022 (95)	Patients with CP (*N* = 1,453; 878; 813 over 3 surveyed periods)	41.7 (13.1)	34.8% M, 65.2% F
Oh, 2021 (96)	Patients with musculoskeletal disorders, COVID-19, and identified as long-term opioid users (*N* = 7,713)	NR	39.5% M, 60.5% F
Prater, 2020 (97)	Underserved patients with chronic spinal pain (*N* = 35)	NR	46% M, 54% F
Tuan, 2021 (100)	Adult patients (*N* = 418,216) with COVID-19; treated with LTOT for CP (*N* = 9,558)	52.1 (17.1) on LTOT; 43.1 (17.6) not on LTOT	39.4% M, 60.6% F on LTOT; 49% M, 51% F not on LTOT

NR, not reported; OUD, opioid use disorder; LTOT, long-term opioid therapy; CP, chronic pain.

## 3. Results

### 3.1. Overall study characteristics

Of the 25 included studies, 36% (*n* = 9) were published in 2020, 52% (*n* = 13) in 2021, and 12% (*n* = 3) in 2022. We identified one article from Pakistan, two from Singapore, one from South Korea, and 16 from the US, with the remaining five focusing on multiple countries. Of the 12 original research articles, only two were outside the US. For marginalized patient populations, articles most frequently focused on race/ethnicity (52%), SES or groups with limited financial capabilities (40%), and the elderly (44%).

### 3.2. Chronic pain, opioid use, and COVID-19 risk

#### 3.2.1. Risk among individuals with chronic pain

COVID-19 infection and mortality rates in the general population are higher among the elderly, racial and ethnic minority groups, low SES groups, people with chronic underlying conditions, and people in assisted living facilities or with limited access to care (3, 9, 81, 82, 85, 89, 96, 98–100). Thus, many individuals with chronic pain are also at high risk for COVID-19 infection as they may be elderly, those with low SES, smokers, those residing in nursing facilities, those with co-occurring chronic diseases, and those with limited access to care (3, 9). Individuals with chronic pain and comorbid conditions (e.g., substance use disorder, asthma, HIV infection, cystic fibrosis, smoking, overweight/obesity, cerebrovascular disease, and type 1 diabetes) are particularly vulnerable to severe COVID-19 infection (9).

#### 3.2.2. Risk among individuals with chronic pain who use opioids

People with chronic pain who use opioids, whether they are on long-acting opioid substitution medication as part of their medication-assisted treatment (MAT) for OUD or on long-term opioid analgesics are at greater risk of morbidity, mortality, healthcare utilization, and severe COVID-19 infection (3, 9, 81, 82, 85, 89, 96, 98–100). Increased risk of morbidity can be attributed to the immunosuppressive mechanism of action (i.e., with steroids and opioid analgesics) and the respiratory depressive mechanism of action (i.e., with opioid analgesics) (89). Within this population, comorbid conditions such as lung disease (e.g., asthma, chronic obstructive pulmonary disease, pulmonary fibrosis, smoking), obesity (i.e., BMI >30), heart disease (i.e., coronary artery disease, cardiomyopathies, heart failure), diabetes (i.e., Type I and Type II), an immunocompromised state (e.g., genetic immunodeficiencies, cancer, HIV, cystic fibrosis), or being of older age further increase risk to complications and comorbidity from COVID-19 infection (9, 82, 85, 98, 99). One study found that compared to controls, patients with chronic pain on LTOT infected with COVID-19 were at increased risk of visiting the emergency room (RR 2.04), being hospitalized, (RR 2.91), requiring intensive care, mechanical ventilation, vasopressor support, and dying within 30 days (100).

#### 3.2.3. Risk among individuals with comorbid chronic pain and OUD

People with chronic pain and OUD are increasingly vulnerable to COVID-19 infection due to the high rates of chronic obstructive pulmonary disease, respiratory depression, and hypoxia associated with opioid misuse (81). Methamphetamine or amphetamine, of which usage has increased among people with OUD, often for self-managing opioid withdrawal, is also known to cause lung injury, pulmonary hypertension, and cardiomyopathy, further amplifying the risk for fatal outcomes of COVID-19 (3). Opioid therapy is considered a risk factor for fatal outcomes among patients with chronic pain, OUD and COVID-19 infection, as overdose can cause respiratory depression and hypoxemia, leading to cardiopulmonary and neurological complications (3). One study from South Korea found patients with musculoskeletal disorders who used strong opioids compared to non-users had a significantly increased in-hospital mortality rate (Odds ratio: 12.73, 95% confidence interval: 2.44–16.64; *p* = 0.002). Prevention of OUD is emphasized, given the increased mortality risk among strong opioid users infected with COVID-19 (96).

### 3.3. COVID-19 disruptions to assessment and diagnosis, ongoing care, and treatment

Disruption to care has been one of the biggest threats for individuals in need of, or already in treatment, for pain management and opioid-related services during the COVID-19 pandemic. The implementation of pandemic-related precautionary measures and shifting priorities toward COVID-19 led to sharp reductions in the assessment, diagnosis and treatment of chronic pain and OUD (89).

#### 3.3.1. Pain management services

Significant reductions, postponements, or cancelations of pain management and other relevant healthcare services were reported globally (3, 78, 79, 81, 84, 86, 88, 90, 98), including decreases in interventional pain techniques and elective surgical procedures, outpatient procedures, patient visits (78, 82, 99), and non-urgent yet vital healthcare services (81). Estimates from a US health system tracker found reductions as high as 86% in elective surgical volume and 15.1% in pain-related prescriptions (3). In lieu of in-person services, clinical visits were predominantly conducted *via* video or telephone. A US study reported 17.7% reductions in in-person clinical volume, 13% reductions in procedure volume, and that 74.5% of all ongoing clinic visits with interventional pain physicians were conducted virtually during the early phases of the pandemic (88). Limited in-person services also contributed to delays in the assessment and diagnosis of chronic pain, as providers were unable to physically examine and evaluate new patients (3, 98), with data from one study reporting a smaller proportion of patients who received pain diagnoses in 2020 than in 2019 (90). Service disruptions were also reported in developing countries, including Egypt, Armenia, Vietnam, Mexico, and India, leaving many patients with chronic pain without access to care. Moreover, available pain management interventions were limited to acute, emergency, and cancer pain management (79).

#### 3.3.2. Social services and social support

Compounding the fear of the COVID-19 disease itself, the implementation of social distancing measures worldwide sparked acute panic, anxiety, depression, obsessive behavior, hoarding, paranoia, and PTSD (3, 9, 81, 94), while also drastically curtailing avenues of social connection that promote recovery. Closures or reductions in housing and other social services and treatment centers markedly affected marginalized communities. Opioid treatment program facilities providing medical treatment and other necessary social support groups and services for vulnerable populations were largely disrupted and moved online (81). Individuals with chronic pain and OUD who recently initiated treatment, but had not yet stabilized on MAT, were particularly vulnerable due to reduced psychosocial functioning and a need for multiple levels of social support to address their increased risk-taking behaviors and multiple drug dependencies.

One US survey study reported that among the 12.7% of the total sample receiving mental health treatment for chronic pain, 30% reported appointment cancelations without future sessions scheduled, 20% reported postponement, and 40% reported that their services were converted to telehealth (94). Given the high prevalence of mental health disorders among patients with chronic pain, mental health implications of the pandemic comprise elevated levels of psychiatric symptoms, stress linked with isolation measures, and an urgency for delivering screenings and interventions (9).

#### 3.3.3. Contributing factors to service delays and disruptions

Reasons for service delays and disruptions include COVID-19-related health anxiety and social-distancing measures as barriers to non-urgent healthcare (79, 80, 88). Patients’ fear of COVID-19 infection was often a barrier to pursuing in-person services (79). Among providers, primary cited reasons for limiting in-person patient interactions were public safety (68.8% of respondents), concerns regarding patient safety (61.9%), staff safety (59.6%), personal/family safety (49.2%), corticosteroid concerns (42.7%), overwhelming the health care system (42.3%), and limited personal protective equipment (32.3%) (88). Extrinsic factors limiting in-person clinic encounters included administrative requirements (46.5%) and patients opting out of in-person visits (52.7%). Providers reported reducing or stopping in-office visits for high-risk patients with chronic pain who met the following criteria: over the age of 65 (89.6%), immunocompromised (83.5%), cardiovascular disease (83.9%), pulmonary diseases other than asthma (87.7%), asthma (83.5%), and active smoking (76.5%) (88). Furthermore, pain clinics serving low-income populations and in developing nations reported staff shortages due to isolation measures and staff being recruited to intensive care and isolation units to prioritize patients with COVID-19 infections (79, 80).

#### 3.3.4. Opioid prescription trends

The proportion of patients with chronic pain receiving opioids varied across the different phases of the pandemic (86, 88, 90, 92). Patients were more likely to receive longer opioid prescriptions and higher doses in the early pandemic period (e.g., March through June 2020) than in 2019 in the US (90). However, this trend reverted to 2019 levels as the pandemic progressed, which may be attributed to state closures and early re-openings later into 2020 (90). In another study, among pain providers surveyed for changes in opioid prescriptions, 65.0% did not report changes, 28.8% reported significant increases, and 6.2% reported significant decreases (*p* < 0.001) (88). Literature suggests that while adaptive during the early shutdowns, these prescribing responses (e.g., excessive exposure to opioids, higher doses, longer prescriptions) may have increased patient risk of future misuse or dependence. Patients may have been newly introduced to opioids (e.g., those with acute pain) or had their dosage increased during a period of limited access to nonpharmacologic therapy (90). Still, other settings (e.g., Singapore in an elderly population) reported avoidance of new opioid prescriptions, given the role of opioids in increasing the risk of COVID-19 infection and morbidity (86).

Two pre-post studies compared trends in prescription opioid usage among marginalized groups, before and during the pandemic (91, 92). While the first study did not find significant changes in pharmacological treatments (NSAIDs or opioids) for low back pain overall, increasing age was positively associated with opioid usage during the pandemic after controlling for demographic factors (multivariate OR = 1.23; 95% CI, 1.05–1.44). Trends toward more frequent use were observed among older and African American participants (92). In the follow-up study, opioid usage remained unchanged during the pandemic (OR, 1.00; 95% CI, 0.58–1.73; *p* > 0.99) (91).

##### 3.3.4.1. Access to prescription opioids for pain

Several articles reported reductions in access to prescription opioids for chronic pain. Patients from underserved communities faced treatment barriers before the pandemic, but the COVID-19 pandemic and structural stigma around opioids exacerbated inaccessibility (3, 78, 79, 81, 86, 87, 94).

Significant declines in the availability of pain medications since the beginning of the pandemic were reported (3, 81, 86). In one study, 40.0% of patients receiving opioid therapy reported that the pandemic impacted their prescriptions, and 19.7% reported limited access due to the pandemic. Among those with access, 21.4% reported concerns about future prescription access (94). Other articles emphasized the underutilization of opioids, especially in developing countries, due to a supply shortage of imported opioids from manufacturers overseas and travel bans (79, 81, 87). As mobility restrictions during the pandemic have been mostly heterogenous globally in terms of duration, intensity, and timing, the initial disruption of usual drug supply chains has led to a short supply of illicit drugs and favored synthetic opioids, affecting patients with chronic pain who require medication for their treatment (81, 87). Since the onset of the pandemic, recent data suggests quick recovery in drug markets; notably, heroin markets (saturated with illicit synthetic opioids such as fentanyl) have continued to expand, further increasing morbidity and mortality (87).

Transportation was cited as another barrier to treatment among marginalized patients with chronic pain, particularly in rural and low-income areas. In some clinics, opioids were often unavailable for home delivery, requiring patients to self-collect from hospital pharmacies (86). Especially in developing countries with limited public transportation options and where hospitals were transformed into isolation hospitals, patients were often unable to travel to farther medical facilities, whether it be due to disability or socioeconomic factors (78). Patients faced isolation measures, transportation restrictions, limited home delivery options, challenges making arrangements to collect their medications, and additional disability or financial barriers (78, 79). This has notably affected patients with low SES or from low-resource settings, suggesting that medications be made at a local level or that systems are developed to deliver medications at doorstep.

### 3.4. Biopsychosocial consequences of the COVID-19 pandemic

#### 3.4.1. Pain and functional outcomes

Lockdown and quarantine orders resulted in increases in inactivity for patients with chronic pain, leading to deconditioning and worsened pain and functional outcomes (81, 85, 91, 92, 94, 97). Worse pain-related and functional outcomes were consistently reported for racial and ethnic minority groups (e.g., Black patients) (81, 91, 92, 94), and less consistently for older or female patients (92). Black and non-Hispanic patients with chronic pain reported greater disruptions in mood, sleep quality (both of which factors exacerbate pain), worse pain interference, and nonsignificant worsening in pain intensity compared to white participants (91, 94, 97). The pandemic was also correlated with an increase in pain perception and a lack of improvement in pain and functional disability in an underserved population (97).

#### 3.4.2. Psychological outcomes

Literature emphasized the consequences of untreated chronic pain and high risk of poor psychological outcomes. Pandemic-related stressors could increase the frequency and intensity of pain symptoms, anxiety, depression, PTSD, leading to functional decline (3, 85, 92). When denied assessment and treatment, patients experienced significant changes in pain intensity levels, emotional suffering, and disability, with 50% of individuals reporting an increase in depressive symptoms, and 35% reporting suicidal ideation (3). Patients from marginalized groups (i.e., Hispanic, lower education and income) experienced higher depressive symptoms and opioid misuse behaviors than the general population (95). Additional factors, such as sudden unemployment or under-employment during the pandemic, contributed to worse psychological outcomes in vulnerable populations (3).

In particular, loneliness due to social isolation measures emerged as an issue that worsened throughout the pandemic (81, 86, 89). Loneliness could exacerbate mental health and substance use issues and increase health care utilization, especially among the elderly and other vulnerable populations living alone with home quarantine orders (86, 89).

#### 3.4.3. Untreated pain, increased risk

Inadequate or unavailable health services, treatment delays, closure of treatment facilities, social isolation, loneliness, and fear of contracting COVID-19 heightened the risk of relapse and overdose (3, 9, 81, 82, 86, 89). Individuals unable to get diagnosed and begin treatment could suffer from significant psychosocial impact, increasing disability, and prescription and illicit drug abuse (3). Existing patients were forced to manage their pain at home with limited telemedicine support, further increasing risk (3, 82, 86). When faced with poorly addressed chronic pain states and withdrawal symptoms, patients may seek out alternative treatments or engage in potentially high-risk behaviors such as escalating opioid use and abuse of alcohol and illicit drugs (3, 81, 89).

Prescription opioids also may be used, consciously or not, to cope and manage symptoms unassociated with pain, such as for pre-existing mental and physical health issues (e.g., depression, anxiety, sleep issues) exacerbated by the pandemic. Such usage can also adversely affect pain-related outcomes. Psychological stress is causally linked to chronic pain and amplifies opioid cravings among those with a history of misuse, increasing the risk for opioid abuse and relapse (9). Usage of illicitly manufactured fentanyl, alcohol, and benzodiazepines may further result in a concomitant increase in overdose fatalities (81).

##### 3.4.3.1. Housing insecurity

Patients with chronic pain and OUD with housing insecurities, for whom opioid overdoses have been a leading cause of death, are particularly vulnerable to adverse health outcomes (81). During the pandemic, isolation measures presented a significant barrier to harm reduction. This particular group faced particular risks of viral infection as well. People experiencing homelessness who were relocated for quarantine were more likely than the general public to be housed in close quarters, often living in crowded groups in shelters, hotels, or areas with limited airflow and poor hygiene. Those experiencing homelessness or incarceration often live in congregate settings (e.g., encampments or abandoned buildings) without regular access to basic hygiene and hand washing facilities, where the risk of COVID-19 virus transmission is great (81). Due to social distancing, people were also less likely to be available to administer naloxone, an opioid reversal agent, leading to fatal overdoses (81).

#### 3.4.4. Opioid-related deaths

Opioid-related deaths during the pandemic significantly increased in the US, especially in marginalized communities (3, 81, 87). The CDC reported record numbers of over 92,000 drug overdoses in 2020 and over 100,000 in 2021, the highest number of overdose deaths ever recorded in a 12-month period (3, 81, 101). Overdose-related deaths increased by 40.0% between 2019 and 2020, and by 28.5% between 2020 and 2021 (81, 101). The age-adjusted rates of fatal opioid overdose were highest among Non-Hispanic American Indians or Alaska Natives (28.0 per 100,000 population) and non-Hispanic Black or African Americans (26.8 per 100,000), surpassing that of non-Hispanic whites (25.8 per 100,000) in 2020 (87). Opioid-related deaths from natural, semi-synthetic and synthetic opioids all increased significantly in the 12-month period ending in April 2021, and were notably driven by synthetic opioids (3).

### 3.5. Innovations and modifications to standards of care during the pandemic

#### 3.5.1. Opioid prescribing and dispensing regulations

Modifications to opioid prescribing and dispensing regulations and reimbursements to accommodate the expansion of telemedicine services and reduce in-person encounters have been observed globally, including in India, Mexico, Egypt, some Eastern European countries, several Latin countries, and the US (79, 82, 87). Brazil, for example, approved legislature allowing for digitally validated, electronic prescriptions of controlled drugs, simple prescriptions, and medical reports (82). In the US, federal requirements for opioid prescriptions were relaxed, increasing the ability for patients to receive methadone take-home doses for OUD and waiving the requirement that initial buprenorphine dosing be in-person (87). Other modifications allowed for repeating fillings of opioid prescriptions for more extended periods than usual (79, 82).

#### 3.5.2. Workflow adaptations and community collaborations

Medical entities adopted new strategies and innovations to expand care accessibility during the pandemic, among which community collaboration and integration across healthcare sectors are promising for marginalized communities (80, 86). Collaboration between community healthcare teams, primary care physicians, hospital pharmacists, and social services allowed for improvements in care (e.g., home delivery of medication refills) for vulnerable patients (i.e., elderly, had impaired mobility, and/or had multiple comorbidities). To ensure continuity of care, referrals were made to community nursing and palliative home-care teams who could closely monitor opioid stock and usage while helping to reduce emergency visits and hospital admissions (80, 86).

#### 3.5.3. Innovations in the delivery of medication-assisted treatment for OUD

Innovations such as technologically-aided time-release pill lockboxes emerged for take-home dosing for patients with chronic pain and OUD. One study evaluated using an electronic and cellular-enabled pillbox for managing methadone among patients (44.0% identified as African American and 57.7% were unemployed or on disability) with moderate and persistent pain and OUD (83). The study reported high satisfaction from patients, with 86.3% reporting they would choose the pillbox again and 95.4% reporting they would recommend it to others, and no observed evidence of actual or attempted methadone diversion.

#### 3.5.4. Telemedicine expansion

The introduction of telemedicine led to the opening of telemedicine pain clinics (e.g., US, Pakistan) and the adoption of video consultations in hospitals and clinics globally, with mixed results for marginalized populations (78, 84, 88, 89).

Given that patients with chronic pain often have mobility and transportation problems, expanding clinical appointments through telehealth can potentially improve access to care (9, 84, 98, 99). In a study evaluating pandemic-related adaptations made by an interdisciplinary pain team to deliver virtual services, patients reported satisfaction, easy access to appointments, and appreciation for the interdisciplinary approach (84). Given the absence of suitable alternatives and their relatively low risks, telehealth also has facilitated promising non-opioid strategies such as mobile health technology *via* web-based or other forms of remote communication to disseminate psychological treatments (e.g., cognitive behavioral therapy, mindfulness therapy, and Acceptance and Commitment Therapy) (9).

Despite innovations in telemedicine as a viable alternative to face-to-face consults, not everyone can benefit. Patients with chronic pain who use opioids, particularly those who are socioeconomically disadvantaged, underserved, elderly, and less educated may not have access to or skills required to use technology in telehealth (80, 98). Poor internet services, limited broadband access, and technological challenges were frequently cited as barriers globally (78, 84, 86).

Patients with technological access to telehealth shared concerns over confidentiality, quality of patient-provider interactions, and the notable differences in the quality of different platforms (video vs. telephone) (98). In a US study, patients reported a lack of clarity on treatment plans, dissatisfaction with visit structures (i.e., telehealth appointments were too long), and preferred in-person appointments (84). In Pakistan, barriers within clinic efforts to reach patients with limited resources by phone included patients’ difficulty understanding medication names and scheduling, language barriers, and concerns about maintaining privacy (78). In Egypt, while patients could readily reach physicians by phone, not having a smartphone with good camera quality was a barrier to acquiring needed opioid prescriptions (79). Those who were older and less ‘tech savvy’ expressed hesitation over telehealth, and would prefer telephone over video platforms or in-person consultations altogether (86). The combined challenges of using technology, living alone with little to no provision of medication, and an insufficient supply of medication and telehealth appointments contributed to inadequate and disrupted care (81, 86).

Some healthcare systems lacked infrastructural support for telehealth, especially when telemedicine had not yet been integrated into pain management services before the pandemic (86, 98). In addition to reductions in in-person activity, some clinics endured major staffing cuts or lacked appropriately trained staff (i.e., nurse practitioners, general practitioners) in their new telemedicine treatment centers. Reduced capacity ultimately limited the types of telemedicine services offered (78, 93).

Given that pain management, especially education on the assessment and treatment of OUD, is often underrepresented in clinical educational curricula, adaptations made during the pandemic also contributed to increased burden of care for clinicians. Compounding fears of COVID-19 infection, increased stress, symptoms of depression and anxiety, sleep disturbances, and increased burnout rates among clinicians, nurses, and frontline workers were cited globally, all of which could potentially impair clinical judgment (3, 85, 93). While clinicians ultimately sought to balance the risk of harm with minimizing pain in vulnerable pain populations, reallocation of healthcare resources and altered work natures (i.e., shifting to telehealth) were cited as barriers to efficacious pain treatments (80, 93).

Telemedicine usage also varied based on geographic location. In the US, accessibility to telehealth emerged as a prominent issue in rural and underserved urban regions where broadband is unavailable, thus affecting people with chronic pain living in poverty, experiencing homelessness, and racial health inequities (89). One study reported that telemedicine visits in rural (17.2%, *p* = 0.008) and suburban (21.9%, *p* = 0.026) areas were performed less often compared with those performed in urban areas (34.3%) (88).

Finally, not all patients were eligible for virtual consultations (86). Telemedicine was often limited to follow-up patients with stable pain conditions. New case consults, patients with worsening pain conditions and patients on opioid medications were usually required to be in-person (80).

## 4. Discussion

This scoping review integrates the published literature regarding the concurrent epidemics of chronic pain and opioids in the context of the COVID-19 pandemic and its impact on marginalized groups. The pandemic has affected treatment of chronic pain and OUD, burdening marginalized groups and revealing further disparities in resources, access to pain and opioid-related services, and health outcomes. Most articles were from the US and other high-income nations, highlighting the relative paucity of research conducted concerning marginalized groups and the need to prioritize vulnerable populations when researching any of these topics.

Isolation measures and dramatic reductions in in-person clinical encounters to mitigate the spread of COVID-19 left many patients overburdened with their pain, treatment delayed, and without access to necessary medication or formal support networks. Frequently reported issues in treatment accessibility and disruptions in ongoing care (e.g., cancelation of appointments, conversion to telehealth, limited or lack of access to prescription opioids) compounded existing inequities for patients with chronic pain who use opioids, particularly those from low-income communities, racial and ethnic minority groups, the elderly, people with disabilities, and those experiencing homelessness (73).

Additional psychosocial stressors from the pandemic contributed to psychological stress, resulting in increasing pain perception and symptoms among individuals of Black and non-Hispanic origin (94). These findings are in line with recent studies highlighting disproportionately higher rates of infection, disability, and death from COVID-19 among people of color, in addition to those over 65 and living below the poverty level (102–106). Patients from marginalized groups additionally have higher rates of underlying chronic and medical conditions, may be overrepresented in essential front-line jobs, and have less access to quality healthcare - all of which increase virus exposure and exacerbate health outcomes (107, 108). Socioeconomic disparities in pain severity, interference, and mental health symptoms were significant, highlighting the need to address structural inequality for those with limited access to resources to promote effective pain management (95).

Despite numerous challenges during the COVID-19 pandemic, the opportunities ahead call for action and innovative solutions. Bold efforts in public policy, critical research, and new partnerships are needed to minimize the aggravating effects that the pandemic may have had on this complex public health crisis.

### 4.1. Telemedicine

The COVID-19 pandemic has driven telemedicine’s rapid adoption, providing a blueprint for embracing virtual care and telehealth after the pandemic. This has allowed medical professionals, especially those with formerly low access in developing countries (79), to benefit from online clinical resources and incorporate telemedicine into pain management for the first time. Previous studies suggest telehealth’s potential to dramatically expand access to care due to its convenience, wider acceptability among consumers, and decreased cost (109–111). This is important for marginalized communities with greater access barriers (e.g., transportation, conflicts with employment). While implementing telehealth is promising for some patients (i.e., those with stable pain conditions), health systems must continue improving access. Although only a handful of articles were from outside of the US, they provided a lens into some of the health system challenges faced in developing nations and low-income settings, from chronic shortages of trained staff, effective treatments, to built infrastructure to support the implementation of telehealth services. A recent scoping review similarly found that infrastructure and regulatory barriers were most significant to wider telemedicine use in LMICs during the pandemic, but more research is needed in this area, taking into account the material and interactional contexts where telehealth is deployed.

As symptom management can be dire for patients waiting for the reopening of in-person primary care services, strategies to ensure continuity of care may include conducting home visits (when telehealth is not an option), community-based multidisciplinary care, and employing community teams to mobilize volunteers who can support patients in technology literacy and access mobile devices. In response to frequent patient concerns over prescription opioid access for pain and the increasing rate of opioid overdoses in the general population throughout the pandemic, we recommend clinicians develop safety plans for patients who are long-term opioid users for their pain to prevent withdrawal symptoms and increased pain when inevitably faced with reduced medication access. Further, patients who receive opioid therapy for chronic pain should be supplied naloxone as opioid overdoses can occur by accident, with household members trained in its administration (81).

Clinicians seeking comprehensive assessment have found themselves constrained in assessment and prescription practices on telehealth platforms, causing clinician judgment and discretion to play an atypically prominent role in treatment. Thus, training should also be provided to nurses and physicians to enhance video consultations, such as being well-versed in questions that can facilitate an accurate assessment of the patient’s overall home environment or in non-verbal cues (86). Another contributing factor may be the lack of appropriate guidelines that maximize accountability and reduce liability (112, 113). A limited number of studies on telehealth have assessed for harm thus far, highlighting the need for research on patient health outcomes from this relatively new method of interfacing (114–117). We recommend rigorous research into this area and for entities like SAMHSA to lay out guidelines (e.g., on take-home dosing, telehealth provision) to support providers and improve health outcomes. Longitudinal studies are also needed to understand the pandemic’s short-and long-term impact, as well as the potential benefits and harms of telehealth on pain-and opioid-related assessment and treatment by providers, and patients’ experiences, coping, and functioning.

### 4.2. Mental health

Another aspect highlighted by the pandemic is the continued need to use the biopsychosocial approach in treatment, particularly psychological monitoring during periods of a health crisis when working with marginalized groups. Patients awaiting treatment for chronic pain have reported high symptom burden, increased pain severity, deteriorating health-related quality of life, and more severe depression and suicidal thinking (118, 119). Older adults with chronic pain in isolation, particularly those with cognitive decline or dementia, may feel more anxious, angry, stressed, agitated, and withdrawn, and require extra psychosocial support (120). Thus, clinicians can include multidisciplinary pain interventions and other remotely supported services (e.g., mobile health applications) as part of regular pain management.

### 4.3. Federal and state-level initiatives

With the closure of treatment facilities, patients with chronic pain and OUD faced barriers to receiving methadone or buprenorphine. Public policies must continue championing access to convenient and safe evidence-based treatments for chronic pain and opioid dependence and ensure equal access to both methadone and buprenorphine, particularly for marginalized low-income and racial and ethnic minority communities. These communities are particularly impacted by the severe consequences of untreated opiate dependence, such as high rates of HIV infection, overdose, and incarceration. Moreover, these areas have been historically vulnerable to outsized impact during disasters and emergencies due to federal and state neglect in establishing necessary socioeconomic safety nets (121, 122). Federal and state-level initiatives to improve treatment outcomes are essential and have already been proven to significantly impact local treatment utilization in certain communities, as seen recently in New York City (123, 124). Top priorities are increasing public sector access and ensuring that PCPs in socioeconomically disadvantaged communities are equipped to handle the assessment and treatment of chronic pain and OUD. Given the recent success of MAT, public officials must continue implementing institutional incentives for public clinics to train and certify PCPs in buprenorphine management. As PCPs reportedly quickly hit limited caps on waivers for buprenorphine prescriptions (125, 126), increasing caps on buprenorphine prescription waivers would be an appropriate direction for the DEA and SAMHSA. Suggested alternatives include incorporating MAT-prescription training in medical school (127). As a particular area of focus, stigma-reduction training programs for physicians, nurses, and physician assistants are recommended to expand the availability of MAT prescribers in public sectors (128).

Since March 2020, in response to social distancing guidelines, the US government eased regulations to allow flexibility in OUD treatment; providers can now initiate buprenorphine treatment in telemedicine (98). More than half the states have received a blanket waiver so that patients can also obtain 28 days’ worth of medicine for use at home. However, according to recent data from The Pew Charitable Trusts, only 24 states plan to maintain take-home methadone policies post-pandemic (129). Federal and state agencies should anticipate and address the potential adverse effects of prescribing policy changes on patient subpopulations who might be harmed by them and preserve these flexibilities by allowing patients with chronic pain and OUD to receive their federally approved medications.

The available evidence shows that restricting opioid prescriptions will not eliminate the epidemic. Prescription opioid overdose deaths are declining but have come hand in hand with significant reductions in access to opioid prescriptions, resulting in the undertreatment of pain in marginalized populations. Findings from our review align with those from previous studies on the poor management of pain and lack of access to prescription opioids and treatment in underserved communities, especially in LMICs (87, 130). The pandemic further highlights the importance of economic and social issues in the opioid epidemic, linking the role of opioids as refuge from physical and psychological trauma, systemic marginalization of communities, and isolation (3). By criminalizing the use of opioids, we miss out on treating the larger population of people worldwide with debilitating conditions who require pain management to function in daily life. Instead, employing strategies and measures for the prevention and management of pain (i.e., increased attention toward advocacy, availability of prescription opioids, and diverse treatment options) in marginalized communities and addressing the opioid crisis with a multidisciplinary and multidimensional approach at a structural level is urgently needed.

### 4.4. Limitations

Due to the recent and evolving nature of the pandemic, this scoping review is limited by the relative paucity of primary studies on our topic and therefore may not be exhaustive. Given the nature of this review, only a descriptive analysis and no risk of bias analysis was performed. Future research should focus on the impact of health system changes throughout the COVID-19 pandemic as it has come in successive “waves,” and efficacy studies to assess the effectiveness and cost-effectiveness of telemedicine during the pandemic on marginalized groups.

### 4.5. Conclusion

The COVID-19 pandemic has exposed and exacerbated existing public health crises globally. To tackle endemic structural inequalities that have led to the rise of chronic pain and opioid dependencies, we must capitalize on the qualities of strength and resiliency that communities throughout the world have demonstrated throughout the pandemic. Our findings may inform approaches to address social determinants of health and patient needs to support marginalized groups in emergent crises. Although the road to recovery is fraught with difficulty, simultaneous attention to the multiple crises of COVID-19, chronic pain, and opioids can present new opportunities for communities to create resources, implement policies, and develop innovations in treating pain.

## Contributions to the literature

The COVID-19 pandemic has exacerbated ongoing public health crises globally, including chronic pain and opioid use. This article discusses how the differential distribution of pain burden across marginalized groups has exacerbated these crises. Our findings may inform approaches to address social determinants of health and patient needs to support marginalized groups in emergent crises. Although the road to recovery is fraught with difficulty, simultaneous attention to the multiple crises of COVID-19, chronic pain, and opioids can present new opportunities for communities to create resources, implement policies, and develop innovations in the treatment of pain.

## Data availability statement

The original contributions presented in the study are included in the article/supplementary material, further inquiries can be directed to the corresponding author.

## Author contributions

KC conceptualized the project and edited and revised. KC, EZ, KL, and DH contributed to the design, data collection, analysis of the project and drafted sections of the manuscript. LY edited and made substantial revisions to the final version of the manuscript. All authors contributed to the article and approved the submitted version.

## Funding

KC was supported by the Li Ka Shing Family Fellowship from the Li Ka Shing Foundation Initiative for Global Mental Health and Department of Social and Behavioral Sciences, School of Global Public Health, New York University (PI: LY, PhD).

## Conflict of interest

The authors declare that the research was conducted in the absence of any commercial or financial relationships that could be construed as a potential conflict of interest.

## Publisher’s note

All claims expressed in this article are solely those of the authors and do not necessarily represent those of their affiliated organizations, or those of the publisher, the editors and the reviewers. Any product that may be evaluated in this article, or claim that may be made by its manufacturer, is not guaranteed or endorsed by the publisher.

## References

[1] WHO (2022). Weekly epidemiological update on COVID-19-18 [Internet]. Available at: https://www.who.int/publications/m/item/weekly-epidemiological-update-on-covid-19---18-may-2022 (Accessed May 22, 2022).

[2] Webb Hooper M Nápoles AM Pérez-Stable EJ. COVID-19 and racial/ethnic disparities. JAMA. (2020) 323:2466–7. doi: 10.1001/jama.2020.8598, PMID: 32391864 PMC9310097

[3] Manchikanti L Vanaparthy R Atluri S Sachdeva H Kaye AD Hirsch JA. COVID-19 and the opioid epidemic: two public health emergencies that intersect with chronic pain. Pain Ther. (2021) 10:269–86. doi: 10.1007/s40122-021-00243-2, PMID: 33718982 PMC7955940

[4] GBD 2017 Disease and Injury Incidence and Prevalence Collaborators Abate D Abate KH Abay SM Abbafati C Abbasi N. Global, regional, and national incidence, prevalence, and years lived with disability for 354 diseases and injuries for 195 countries and territories, 1990–2017: a systematic analysis for the global burden of disease study 2017. Lancet. (2018) 392:1789–858. doi: 10.1016/S0140-6736(18)32279-7, PMID: 30496104 PMC6227754

[5] Ritchie H Roser M. (2019). Drug use. Our world in data [internet]. Available at: https://ourworldindata.org/drug-use (Accessed June 04, 2022).

[6] United Nations: Office on Drugs and Crime. World drug report 2021 [internet]. United Nations: Office on drugs and Crime. (2021). Available at: https://www.unodc.org/unodc/en/data-and-analysis/wdr2021.html (Accessed May 22, 2022).

[7] WHO (2019). International Classification of Diseases (ICD) [Internet]. Available at: https://www.who.int/standards/classifications/classification-of-diseases (Accessed May 22, 2022).

[8] Volkow ND McLellan AT. Opioid abuse in chronic pain—misconceptions and mitigation strategies. N Engl J Med. (2016) 374:1253–63. doi: 10.1056/NEJMra1507771, PMID: 27028915

[9] Cohen SP Baber ZB Buvanendran A McLean BC Chen Y Hooten WM. Pain management best practices from multispecialty organizations during the COVID-19 pandemic and public health crises. Pain Med. (2020) 21:1331–46. doi: 10.1093/pm/pnaa127, PMID: 32259247 PMC7184417

[10] Krausz RM Westenberg JN Ziafat K. The opioid overdose crisis as a global health challenge. Curr Opin Psychiatry. (2021) 34:405–12. doi: 10.1097/YCO.0000000000000712, PMID: 33901060

[11] Centers for Disease Control and Prevention, National Center for Injury Prevention and Control. (2021). Understanding the Epidemic [Internet]. CDC. Available at: https://www.cdc.gov/opioids/basics/epidemic.html (Accessed June 09, 2022).

[12] DeWeerdt S. Tracing the US opioid crisis to its roots. Nature. (2019) 573:S10–2. doi: 10.1038/d41586-019-02686-2, PMID: 31511672

[13] Degenhardt L Grebely J Stone J Hickman M Vickerman P Marshall BDL. Global patterns of opioid use and dependence: harms to populations, interventions, and future action. Lancet. (2019) 394:1560–79. doi: 10.1016/S0140-6736(19)32229-9, PMID: 31657732 PMC7068135

[14] Reuben DB Alvanzo AAH Ashikaga T Bogat GA Callahan CM Ruffing V. National Institutes of Health pathways to prevention workshop: the role of opioids in the treatment of chronic pain. Ann Intern Med. (2015) 162:295–300. doi: 10.7326/M14-2775, PMID: 25581341

[15] CBO (2022). The Opioid Crisis and Recent Federal Policy Responses|Congressional Budget Office [Internet]. Available at: https://www.cbo.gov/publication/58221 (Accessed October 03, 2023).

[16] CDC (2020). Congressional Budget Office, using information from the CDC WONDER database, Centers for Disease Control and Prevention, National Center for Health Statistics, “About Multiple Cause of Death 1999–2020” [Internet]. Available at: https://wonder.cdc.gov/mcd-icd10.html (Accessed October 03, 2023).

[17] Jenkins RA. The fourth wave of the US opioid epidemic and its implications for the rural US: a federal perspective. Prev Med. (2021) 152:106541. doi: 10.1016/j.ypmed.2021.106541, PMID: 34482994

[18] CDC. NVSS-Mortality Data [Internet]. (2023). Available at: https://www.cdc.gov/nchs/nvss/deaths.htm (Accessed October 03, 2023).

[19] Jackman RP Purvis JM Mallett BS. Chronic nonmalignant pain in primary care. AFP. (2008) 78:1155–62. 19035063

[20] El-Metwally A Shaikh Q Aldiab A Al-Zahrani J Al-Ghamdi S Alrasheed AA. The prevalence of chronic pain and its associated factors among Saudi Al-Kharj population; a cross sectional study. BMC Musculoskelet Disord. (2019) 20:177. doi: 10.1186/s12891-019-2555-7, PMID: 31027485 PMC6485157

[21] Zelaya CE Dahlhamer JM Lucas JW Connor EM. Chronic pain and high-impact chronic pain among U.S. adults, 2019. NCHS Data Brief. (2020) 390:1–8. PMID: 33151145

[22] Cohen SP Vase L Hooten WM. Chronic pain: an update on burden, best practices, and new advances. Lancet. (2021) 397:2082–97. doi: 10.1016/S0140-6736(21)00393-7, PMID: 34062143

[23] Beam E. VA/DoD Clinical Practice Guideline for Opioid Therapy for Chronic Pain. Washington, DC: United States Department of Veterans Affairs (2017).

[24] Von Korff M Kolodny A Deyo RA Chou R. Long-term opioid therapy reconsidered. Ann Intern Med. (2011) 155:325–8. doi: 10.7326/0003-4819-155-5-201109060-00011, PMID: 21893626 PMC3280085

[25] Rosenblum A Marsch LA Joseph H Portenoy RK. Opioids and the treatment of chronic pain: controversies, current status, and future directions. Exp Clin Psychopharmacol. (2008) 16:405–16. doi: 10.1037/a0013628, PMID: 18837637 PMC2711509

[26] Pain Management and the Opioid Epidemic. (2022). Balancing societal and individual benefits and risks of prescription opioid use|the National Academies Press [internet]. Available at: https://nap.nationalacademies.org/catalog/24781/pain-management-and-the-opioid-epidemic-balancing-societal-and-individual (Accessed April 06, 2022). 29023083

[27] Vowles KE McEntee ML Julnes PS Frohe T Ney JP van der Goes DN. Rates of opioid misuse, abuse, and addiction in chronic pain: a systematic review and data synthesis. Pain. (2015) 156:569–76. doi: 10.1097/01.j.pain.0000460357.01998.f1 25785523

[28] CDC Guideline for Prescribing Opioids for Chronic Pain — United States. (2016). MMWR Recomm rep [internet] 2016. Available at: https://www.cdc.gov/mmwr/volumes/65/rr/rr6501e1.htm (Accessed December 14, 2022). 10.15585/mmwr.rr6501e1 26987082

[29] Wasan AD Butler SF Budman SH Fernandez K Weiss RD Greenfield SF. Does report of craving opioid medication predict aberrant drug behavior among chronic pain patients? Clin J Pain. (2009) 25:193–8. doi: 10.1097/AJP.0b013e318193a6c4, PMID: 19333168 PMC2664529

[30] Martell BA O’Connor PG Kerns RD Becker WC Morales KH Kosten TR. Systematic review: opioid treatment for chronic back pain: prevalence, efficacy, and association with addiction. Ann Intern Med. (2007) 146:116–27. doi: 10.7326/0003-4819-146-2-200701160-00006, PMID: 17227935

[31] Nadeau SE Wu JK Lawhern RA. Opioids and chronic pain: an analytic review of the clinical evidence. Front Pain Res. (2021) 2:357. doi: 10.3389/fpain.2021.721357, PMID: 35295493 PMC8915556

[32] Gibson A Degenhardt L Mattick RP Ali R White J O’Brien S. Exposure to opioid maintenance treatment reduces long-term mortality. Addiction. (2008) 103:462–8. doi: 10.1111/j.1360-0443.2007.02090.x, PMID: 18190664

[33] Degenhardt L Randall D Hall W Law M Butler T Burns L. Mortality among clients of a state-wide opioid pharmacotherapy program over 20 years: risk factors and lives saved. Drug Alcohol Depend. (2009) 105:9–15. doi: 10.1016/j.drugalcdep.2009.05.021, PMID: 19608355

[34] Gatchel RJ Peng YB Peters ML Fuchs PN Turk DC. The biopsychosocial approach to chronic pain: scientific advances and future directions. Psychol Bull. (2007) 133:581–624. doi: 10.1037/0033-2909.133.4.581, PMID: 17592957

[35] Schiffer K Schatz E. Marginalisation, Social Inclusion and Health: Experiences Based on the Work of Correlation-European Network Social Inclusion & Health. Amsterdam: Foundation Regenboog AMOC (2008).

[36] Baah FO Teitelman AM Riegel B. Marginalization: conceptualizing patient vulnerabilities in the framework of social determinants of health – an integrative review. Nurs Inq. (2019) 26:e12268. doi: 10.1111/nin.12268, PMID: 30488635 PMC6342665

[37] Hall GCN Yip T Zárate MA. On becoming multicultural in a monocultural research world: a conceptual approach to studying ethnocultural diversity. Am Psychol. (2016) 71:40–51. doi: 10.1037/a0039734, PMID: 26766764

[38] Kawachi I. A glossary for health inequalities. J Epidemiol Community Health. (2002) 56:647–52. doi: 10.1136/jech.56.9.647, PMID: 12177079 PMC1732240

[39] Minority Health and Health Disparities: Definitions and Parameters. (2022). NIMHD. Available at: https://www.nimhd.nih.gov/about/strategic-plan/nih-strategic-plan-definitions-and-parameters.html (Accessed February 08, 2022).

[40] Sevelius JM Gutierrez-Mock L Zamudio-Haas S McCree B Ngo A Jackson A. Research with marginalized communities: challenges to continuity during the COVID-19 pandemic. AIDS Behav. (2020) 24:2009–12. doi: 10.1007/s10461-020-02920-3, PMID: 32415617 PMC7228861

[41] Evans EA Hser YI. The natural history, clinical course, and long-term recovery from opioid use disorders In: Kelly JF Wakeman SE, editors. Treating Opioid Addiction. Cham: Springer International Publishing (2019). 181–96.

[42] van Draanen J Tsang C Mitra S Karamouzian M Richardson L. Socioeconomic marginalization and opioid-related overdose: a systematic review. Drug Alcohol Depend. (2020) 214:108127. doi: 10.1016/j.drugalcdep.2020.108127, PMID: 32650191 PMC7313902

[43] Meghani SH Polomano RC Tait RC Vallerand AH Anderson KO Gallagher RM. Advancing a national agenda to eliminate disparities in pain care: directions for health policy, education, practice, and research. Pain Med. (2012) 13:5–28. doi: 10.1111/j.1526-4637.2011.01289.x, PMID: 22142450

[44] Chuang E Gil EN Gao Q Kligler B McKee MD. Relationship between opioid analgesic prescription and unemployment in patients seeking acupuncture for chronic pain in urban primary care. Pain Med. (2019) 20:1528–33. doi: 10.1093/pm/pny169, PMID: 30184213 PMC7739953

[45] Grant M Rees S Underwood M Froud R. Obstacles to returning to work with chronic pain: in-depth interviews with people who are off work due to chronic pain and employers. BMC Musculoskelet Disord. (2019) 20:486. doi: 10.1186/s12891-019-2877-5, PMID: 31656184 PMC6815386

[46] Phillips CJ. Economic burden of chronic pain. Expert Rev Pharmacoecon Outcomes Res. (2006) 6:591–601. doi: 10.1586/14737167.6.5.591 20528505

[47] Dasgupta N Beletsky L Ciccarone D. Opioid crisis: no easy fix to its social and economic determinants. Am J Public Health. (2018) 108:182–6. doi: 10.2105/AJPH.2017.304187, PMID: 29267060 PMC5846593

[48] Ulirsch JC Weaver MA Bortsov AV Soward AC Swor RA Peak DA. No man is an island: living in a disadvantaged neighborhood influences chronic pain development after motor vehicle collision, and this effect is moderated by common genetic variation influencing HPA axis function. Pain. (2014) 155:2116–23. doi: 10.1016/j.pain.2014.07.025, PMID: 25107859 PMC4197076

[49] Dahlhamer J. (2018). Prevalence of Chronic Pain and High-Impact Chronic Pain Among Adults—United States, 2016. MMWR Morb mortal Wkly rep [internet]. Available at: https://www.cdc.gov/mmwr/volumes/67/wr/mm6736a2.htm (Accessed September 06, 2022). 10.15585/mmwr.mm6736a2 PMC6146950 30212442

[50] Altekruse SF Cosgrove CM Altekruse WC Jenkins RA Blanco C. Socioeconomic risk factors for fatal opioid overdoses in the United States: findings from the mortality disparities in American communities study (MDAC). PLoS One. (2020) 15:e0227966. doi: 10.1371/journal.pone.0227966, PMID: 31951640 PMC6968850

[51] Zedler B Xie L Wang L Joyce A Vick C Kariburyo F. Risk factors for serious prescription opioid-related toxicity or overdose among veterans health administration patients. Pain Med. (2014) 15:1911–29. doi: 10.1111/pme.12480 24931395

[52] NYU. Substance Abuse: Clinical Issues in Intensive Outpatient Treatment-NCBI Bookshelf [Internet]. Available at: https://www-ncbi-nlm-nih-gov.ezproxy.med.nyu.edu/books/NBK64093/ (Accessed September 06, 2022).

[53] Campbell CM France CR Robinson ME Logan HL Geffken GR Fillingim RB. Ethnic differences in the nociceptive flexion reflex (NFR). Pain. (2008) 134:91–6. doi: 10.1016/j.pain.2007.03.035, PMID: 17482362 PMC2683417

[54] Campbell TS Hughes JW Girdler SS Maixner W Sherwood A. Relationship of ethnicity, gender, and ambulatory blood pressure to pain sensitivity: effects of individualized pain rating scales. J Pain. (2004) 5:183–91. doi: 10.1016/j.jpain.2004.02.305, PMID: 15106131

[55] Cintron A Morrison RS. Pain and ethnicity in the United States: a systematic review. J Palliat Med. (2006) 9:1454–73. doi: 10.1089/jpm.2006.9.1454 17187552

[56] Carey TS Garrett JM. The relation of race to outcomes and the use of health care services for acute low back pain. Spine Phila Pa. (1976) 28:390–4. doi: 10.1097/01.BRS.0000048499.25275.51 12590217

[57] Lee WW Burelbach AE Fosnocht D. Hispanic and non-Hispanic white patient pain management expectations. Am J Emerg Med. (2001) 19:549–50. doi: 10.1053/ajem.2001.28038, PMID: 11698999

[58] Balsa AI McGuire TG. Prejudice, clinical uncertainty and stereotyping as sources of health disparities. J Health Econ. (2003) 22:89–116. doi: 10.1016/S0167-6296(02)00098-X, PMID: 12564719

[59] Green CR Anderson KO Baker TA Campbell LC Decker S Fillingim RB. The unequal burden of pain: confronting racial and ethnic disparities in pain. Pain Med. (2003) 4:277–94. doi: 10.1046/j.1526-4637.2003.03034.x, PMID: 12974827

[60] Becker WC Starrels JL Heo M Li X Weiner MG Turner BJ. Racial differences in primary care opioid risk reduction strategies. Ann Family Med. (2011) 9:219–25. doi: 10.1370/afm.1242, PMID: 21555749 PMC3090430

[61] Goedel WC Shapiro A Cerdá M Tsai JW Hadland SE Marshall BDL. Association of Racial/ethnic segregation with treatment capacity for opioid use disorder in counties in the United States. JAMA Netw Open. (2020) 3:e203711. doi: 10.1001/jamanetworkopen.2020.3711, PMID: 32320038 PMC7177200

[62] Staton LJ Panda M Chen I Genao I Kurz J Pasanen M. When race matters: disagreement in pain perception between patients and their physicians in primary care. J Natl Med Assoc. (2007) 99:532–8. 17534011 PMC2576060

[63] Tamayo-Sarver JH Dawson NV Hinze SW Cydulka RK Wigton RS Albert JM. The effect of race/ethnicity and desirable social characteristics on physicians’ decisions to prescribe opioid analgesics. Acad Emerg Med. (2003) 10:1239–48. doi: 10.1197/S1069-6563(03)00494-9, PMID: 14597500

[64] Lo CC Cheng TC. Racial/ethnic differences in access to substance abuse treatment. J Health Care Poor Underserved. (2011) 22:621–37. doi: 10.1353/hpu.2011.0054 21551938

[65] Maly A Vallerand AH. Neighborhood, socioeconomic, and racial influence on chronic pain. Pain Manag Nurs. (2018) 19:14–22. doi: 10.1016/j.pmn.2017.11.004, PMID: 29422123 PMC8895435

[66] Saloner B Cook BL. Blacks and Hispanics are less likely than whites to complete addiction treatment, largely due to socioeconomic factors. Health Aff. (2013) 32:135–45. doi: 10.1377/hlthaff.2011.0983, PMID: 23297281 PMC3570982

[67] Substance Abuse and Mental Health Services Administration (US), Office of the Surgeon General (US). Facing Addiction in America: The Surgeon General’s Report on Alcohol, Drugs, and Health. Washington, DC: US Department of Health and Human Services (2016). 28252892

[68] Javed S Hung J Huh BK. Impact of COVID-19 on chronic pain patients: a pain physician’s perspective. Pain Manag. (2020) 10:5. doi: 10.2217/pmt-2020-0035 32772631 PMC7422723

[69] Ghose R Forati AM Mantsch JR. Impact of the COVID-19 pandemic on opioid overdose deaths: a spatiotemporal analysis. J Urban Health. (2022) 99:316–27. doi: 10.1007/s11524-022-00610-0, PMID: 35181834 PMC8856931

[70] Harvard Medical School. Depression and pain [Internet]. Harvard health (2009). Available at: https://www.health.harvard.edu/mind-and-mood/depression-and-pain (Accessed September 06, 2022).

[71] VPergolizzi J Jr Passik S JA LeQuang Colucci D Taylor R Braffa R. (2018). The risk of suicide in chronic pain patients. Nurs Palliat care [internet]. Available at: https://www.oatext.com/the-risk-of-suicide-in-chronic-pain-patients.php (Accessed September 06, 2022).

[72] Brooks SK Webster RK Smith LE Woodland L Wessely S Greenberg N. The psychological impact of quarantine and how to reduce it: rapid review of the evidence. Lancet. (2020) 395:912–20. doi: 10.1016/S0140-6736(20)30460-8, PMID: 32112714 PMC7158942

[73] Eccleston C Blyth FM Dear BF Fisher EA Keefe FJ Lynch ME. Managing patients with chronic pain during the COVID-19 outbreak: considerations for the rapid introduction of remotely supported (eHealth) pain management services. Pain. (2020) 161:889–93. doi: 10.1097/j.pain.0000000000001885, PMID: 32251203 PMC7172975

[74] Peters M Godfrey C Khalil H Mcinerney P Soares C Parker D. Guidance for the Conduct of JBI Scoping Reviews. Adelaide: JBI (2017).

[75] McGowan J Straus S Moher D Langlois EV O’Brien KK Horsley T. Reporting scoping reviews-PRISMA ScR extension. J Clin Epidemiol. (2020) 123:177–9. doi: 10.1016/j.jclinepi.2020.03.016, PMID: 32229248

[76] Cheraghi-Sohi S Panagioti M Daker-White G Giles S Riste L Kirk S. Patient safety in marginalised groups: a narrative scoping review. Int J Equity Health. (2020) 19:26. doi: 10.1186/s12939-019-1103-2, PMID: 32050976 PMC7014732

[77] The World Bank Group. Countries and Economies [Internet]. The World Bank Group. Available at: https://data.worldbank.org/country/ (Accessed September 06, 2022).

[78] Akhtar AB Durrani RS Ghafoor AUR. The new normal – pain control in COVID era. Anaesthesia Pain Intensive Care. (2020) 24:373–6. doi: 10.35975/apic.v24i4.1307

[79] Ayad MD Fipp AE. Opioid epidemics during the pandemic: further insights to the same story. J of Opioid Manag. (2021) 17:9–12. doi: 10.5055/jom.2021.0609 33735423

[80] Chan DX Lin XF George JM Liu CW. Clinical challenges and considerations in Management of Chronic Pain Patients during a COVID-19 pandemic. Ann Acad Med Singap. (2020) 49:669–73. doi: 10.47102/annals-acadmedsg.2020130, PMID: 33241255

[81] Compton P St. Marie B. Coexisting substance use disorder and chronic pain during COVID-19. Pain Manag Nurs. (2022) 23:17–25. doi: 10.1016/j.pmn.2021.08.011, PMID: 34620549 PMC8418911

[82] de Moraes ÉB Santos Garcia JB de Macedo AJ Daher DV Seixas FL Muniz Ferrari MF. Chronic pain management during the Covid-19 pandemic: a scoping review. Pain Manag Nurs. (2021) 22:103–10. doi: 10.1016/j.pmn.2020.11.010, PMID: 33390355 PMC7706418

[83] Dunn KE Brooner RK Stoller KB. Technology-assisted methadone take-home dosing for dispensing methadone to persons with opioid use disorder during the COVID-19 pandemic. J Subst Abus Treat. (2021) 121:108197. doi: 10.1016/j.jsat.2020.108197, PMID: 33357606 PMC7834258

[84] Edmond SN Currie S Gehrke A Falker CG Sung M Abelleira A. Optimizing interdisciplinary virtual pain care and buprenorphine initiation during COVID-19: a quality improvement study. Pain Med. (2021):pnab348. doi: 10.1093/pm/pnab348 PMC9383145 34940877

[85] El-Tallawy SN Nalamasu R Pergolizzi JV Gharibo C. Pain management during the COVID-19 pandemic. Pain Ther. (2020) 9:453–66. doi: 10.1007/s40122-020-00190-4, PMID: 32840756 PMC7445106

[86] George J Xu Y Nursaadah B Lim S Low L Chan DX. Collaboration between a tertiary pain Centre and community teams during the pandemic. Br J Community Nurs. (2020) 25:480–8. doi: 10.12968/bjcn.2020.25.10.480, PMID: 33030369

[87] Humphreys K Shover CL Andrews CM Bohnert ASB Brandeau ML Caulkins JP. Responding to the opioid crisis in North America and beyond: recommendations of the Stanford–lancet commission. Lancet. (2022) 399:555–604. doi: 10.1016/S0140-6736(21)02252-2, PMID: 35122753 PMC9261968

[88] Joyce AA Conger A McCormick ZL Kendall RW Wagner G Teramoto M. Changes in interventional pain physician decision-making, practice patterns, and mental health during the early phase of the SARS-CoV-2 global pandemic. Pain Med. (2020) 21:3585–95. doi: 10.1093/pm/pnaa294, PMID: 32866247 PMC7499755

[89] Katzman JG Katzman JW. COVID-19 has provided 20/20 vision illuminating our Nation’s health crises. Pain Med. (2021) 22:6–9. doi: 10.1093/pm/pnaa357, PMID: 32986827 PMC7543634

[90] Lee B Yang KC Kaminski P Peng S Odabas M Gupta S. Substitution of nonpharmacologic therapy with opioid prescribing for pain during the COVID-19 pandemic. JAMA Netw Open. (2021) 4:e2138453. doi: 10.1001/jamanetworkopen.2021.38453, PMID: 34889946 PMC8665369

[91] Licciardone JC. Impact of COVID-19 on utilization of nonpharmacological and pharmacological treatments for chronic low back pain and clinical outcomes. J Osteop Med. (2021) 121:625–33. doi: 10.1515/jom-2020-0334, PMID: 33770828

[92] Licciardone JC. Demographic characteristics associated with utilization of noninvasive treatments for chronic Low Back pain and related clinical outcomes during the COVID-19 pandemic in the United States. J Am Board Fam Med. (2021b) 34:S77–84. doi: 10.3122/jabfm.2021.S1.200352, PMID: 33622822

[93] Morgan AR Hendricks MA El Ibrahimi S Hallvik SE Hatch B Dickinson C. COVID-19-related adaptations to the implementation and evaluation of a clinic-based intervention designed to improve opioid safety. DIC. (2021) 10:1–8. doi: 10.7573/dic.2021-7-5, PMID: 34970321 PMC8687093

[94] Mun CJ Campbell CM McGill LS Aaron RV. The early impact of COVID-19 on chronic pain: a cross-sectional investigation of a large online sample of individuals with chronic pain in the United States, April to May, 2020. Pain Med. (2021) 22:470–80. doi: 10.1093/pm/pnaa446, PMID: 33537764 PMC7901854

[95] Mun CJ Campbell CM McGill LS Wegener ST Aaron RV. Trajectories and individual differences in pain, emotional distress, and prescription opioid misuse during the COVID-19 pandemic: a one-year longitudinal study. J Pain. (2022) 23:1234–44. doi: 10.1016/j.jpain.2022.02.005 35272053 PMC8898783

[96] Oh TK Song IA Lee J Eom W Jeon YT. Musculoskeletal disorders, pain medication, and in-hospital mortality among patients with COVID-19 in South Korea: a population-based cohort study. IJERPH. (2021) 18:6804. doi: 10.3390/ijerph18136804, PMID: 34202825 PMC8295800

[97] Prater C Tepe M Battaglia P. Integrating a multidisciplinary pain team and chiropractic Care in a Community Health Center: an observational study of managing chronic spinal pain. J Prim Care Community Health. (2020) 11:215013272095368. doi: 10.1177/2150132720953680 PMC7495928 32909504

[98] Rao PN Jotwani R Joshi J Gulati A Mehta N. Reevaluating chronic opioid monitoring during and after the COVID-19 pandemic. Pain Manag. (2020) 10:353–8. doi: 10.2217/pmt-2020-0063, PMID: 32945238 PMC7505054

[99] Shanthanna H Strand NH Provenzano DA Lobo CA Eldabe S Bhatia A. Caring for patients with pain during the COVID-19 pandemic: consensus recommendations from an international expert panel. Anaesthesia. (2020) 75:935–44. doi: 10.1111/anae.15076, PMID: 32259288 PMC7262200

[100] Tuan WJ Spotts H Zgierska AE Lennon RP. COVID-19 outcomes among adult patients treated with long-term opioid therapy for chronic non-cancer pain in the USA: a retrospective cohort study. BMJ Open. (2021) 11:e056436. doi: 10.1136/bmjopen-2021-056436, PMID: 34836910 PMC8628115

[101] CDC. Drug Overdose Deaths in the U.S. Top 100,000 Annually [Internet]. (2021). Available at: https://www.cdc.gov/nchs/pressroom/nchs_press_releases/2021/20211117.htm (Accessed September 04, 2022).

[102] Guerrero LR Wallace SP. The impact of COVID-19 on diverse older adults and health equity in the United States. Front Public Health. (2021) 9:661592. doi: 10.3389/fpubh.2021.661592, PMID: 34079786 PMC8165264

[103] Padalabalanarayanan S Hanumanthu VS Sen B. Association of State Stay-at-Home Orders and State-Level African American Population with COVID-19 Case rates. JAMA Netw Open. (2020) 3:e2026010. doi: 10.1001/jamanetworkopen.2020.26010, PMID: 33095253 PMC7584926

[104] Oronce CIA Scannell CA Kawachi I Tsugawa Y. Association between state-level income inequality and COVID-19 cases and mortality in the USA. J Gen Intern Med. (2020) 35:2791–3. doi: 10.1007/s11606-020-05971-3, PMID: 32583336 PMC7313247

[105] Mays JC Newman A. Virus is twice as deadly for black and Latino people than whites in N.Y.C. [internet]. The New York times. (2020). Available at: https://www.nytimes.com/2020/04/08/nyregion/coronavirus-race-deaths.html (Accessed September 06, 2022).

[106] SAMHSA. Behavioral Health Equity [Internet]. Available at: https://www.samhsa.gov/behavioral-health-equity (Accessed September 06, 2022).

[107] Nania R. Higher COVID-19 incidence in minority communities [internet]. AARP. Available at: https://www.aarp.org/health/conditions-treatments/info-2020/minority-communities-covid-19.html (Accessed August 06, 2022).

[108] Godoy M Wood D. (2020). What do coronavirus racial disparities look like state by state? [internet]. NPR. Available at: https://www.npr.org/sections/health-shots/2020/05/30/865413079/what-do-coronavirus-racial-disparities-look-like-state-by-state (Accessed September 06, 2022).

[109] Buhrman M Gordh T Andersson G. Internet interventions for chronic pain including headache: a systematic review. Internet Interv. (2016) 4:17–34. doi: 10.1016/j.invent.2015.12.001, PMID: 30135787 PMC6096254

[110] Dávalos ME French MT Burdick AE Simmons SC. Economic evaluation of telemedicine: review of the literature and research guidelines for benefit–cost analysis. Telemed E-Health. (2009) 15:933–48. doi: 10.1089/tmj.2009.0067, PMID: 19954346

[111] de la Torre-Díez I López-Coronado M Vaca C Aguado JS de Castro C. Cost-utility and cost-effectiveness studies of telemedicine, electronic, and Mobile health Systems in the Literature: a systematic review. Telemed E-Health. (2015) 21:81–5. doi: 10.1089/tmj.2014.0053, PMID: 25474190 PMC4312789

[112] McLellan AT. Prescription opioids, overdose deaths, and physician responsibility. JAMA. (2008) 300:2672–3. doi: 10.1001/jama.2008.793, PMID: 19066389

[113] Weaver M Schnoll S. Abuse liability in opioid therapy for pain treatment in patients with an addiction history. Clin J Pain. (2002) 18:S61–9. doi: 10.1097/00002508-200207001-00007, PMID: 12479255

[114] Adamse C Dekker-Van Weering MG van Etten-Jamaludin FS Stuiver MM. The effectiveness of exercise-based telemedicine on pain, physical activity and quality of life in the treatment of chronic pain: a systematic review. J Telemed Telecare. (2018) 24:511–26. doi: 10.1177/1357633X17716576, PMID: 28696152

[115] Keogh E Rosser BA Eccleston C. E-health and chronic pain management: current status and developments. Pain. (2010) 151:18–21. doi: 10.1016/j.pain.2010.07.014, PMID: 20674174

[116] Lalloo C Shah U Birnie KA Davies-Chalmers C Rivera J Stinson J. Commercially available smartphone apps to support postoperative pain self-management: scoping review. JMIR Mhealth Uhealth. (2017) 5:e162. doi: 10.2196/mhealth.8230, PMID: 29061558 PMC5673880

[117] McGeary DD McGeary CA Gatchel RJ. A comprehensive review of telehealth for pain management: where we are and the way ahead: telehealth for pain management. Pain Pract. (2012) 12:570–7. doi: 10.1111/j.1533-2500.2012.00534.x, PMID: 22303839

[118] Kowal J McWilliams LA Péloquin K Wilson KG Henderson PR Fergusson DA. Attachment insecurity predicts responses to an interdisciplinary chronic pain rehabilitation program. J Behav Med. (2015) 38:518–26. doi: 10.1007/s10865-015-9623-8, PMID: 25716120

[119] Lynch ME Campbell F Clark AJ Dunbar MJ Goldstein D Peng P. A systematic review of the effect of waiting for treatment for chronic pain. Pain. (2008) 136:97–116. doi: 10.1016/j.pain.2007.06.018 17707589

[120] Lo Bianco G Papa A Schatman ME Tinnirello A Terranova G Leoni MLG. Practical advices for treating chronic pain in the time of COVID-19: a narrative review focusing on interventional techniques. J Clin Med. (2021) 10:2303. doi: 10.3390/jcm10112303, PMID: 34070601 PMC8198659

[121] Baldacci E Mello L de Inchauste G (2002). Financial crises, poverty, and income distribution. IMF Working Papers [Internet]. Available at: https://www.elibrary.imf.org/view/journals/001/2002/004/article-A001-en.xml (Accessed September 06, 2022).

[122] Blomquist J Verhoeven M Cordoba JP Bouillon C Moser P. Social Safety Nets in Response to Crisis: Lessons and Guidelines from Asia and Latin America. In: Towards Asia’s Sustainable Development: The Role of Social Protection. Paris: OECD. (2002). p. 297–332.

[123] Hansen HB Siegel CE Case BG Bertollo DN DiRocco D Galanter M. Variation in use of buprenorphine and methadone treatment by racial, ethnic, and income characteristics of residential social areas in new York City. J Behav Health Serv Res. (2013) 40:367–77. doi: 10.1007/s11414-013-9341-3, PMID: 23702611 PMC3818282

[124] Hansen H Siegel C Wanderling J DiRocco D. Buprenorphine and methadone treatment for opioid dependence by income, ethnicity and race of neighborhoods in new York City. Drug Alcohol Depend. (2016) 164:14–21. doi: 10.1016/j.drugalcdep.2016.03.028, PMID: 27179822 PMC5539992

[125] Beetham T Saloner B Wakeman SE Gaye M Barnett ML. Access to office-based buprenorphine treatment in areas with high rates of opioid-related mortality: an audit study. Ann Intern Med. (2019) 171:1–9. doi: 10.7326/M18-3457, PMID: 31158849 PMC7164610

[126] Lin LK Simon K Hollingsworth A Saloner B. Association between the number of certified buprenorphine prescribers and the quantity of buprenorphine prescriptions: evidence from 2015 to 2017. J Gen Intern Med. (2019) 34:2313–5. doi: 10.1007/s11606-019-05165-6, PMID: 31313114 PMC6848725

[127] Frank JW Wakeman SE Gordon AJ. No end to the crisis without an end to the waiver. Subst Abus. (2018) 39:263–5. doi: 10.1080/08897077.2018.1543382, PMID: 30676296

[128] Haffajee RL Bohnert ASB Lagisetty PA. Policy pathways to address provider workforce barriers to buprenorphine treatment. Am J Prev Med. (2018) 54:S230–42. doi: 10.1016/j.amepre.2017.12.022, PMID: 29779547 PMC6330240

[129] PEW. Most States Eased Access to Opioid Use Disorder Treatment During the Pandemic [Internet]. Available at: https://pew.org/3ayPrSD (Accessed July 06, 2022).

[130] Morriss WW Roques CJ. Pain management in low-and middle-income countries. BJA Educ. (2018) 18:265–70. doi: 10.1016/j.bjae.2018.05.006, PMID: 33456843 PMC7807826

